# Studying and Simulating the Impact of Plasma Activated Water (PAW) on *Salmonella* in Almond Products: A Molecular Dynamics Investigation

**DOI:** 10.1002/fsn3.72094

**Published:** 2026-07-07

**Authors:** Mohammad Javad Aarabi, Sajad Rostami, Bahram Hosseinzadeh Samani

**Affiliations:** ^1^ Department of Mechanical Engineering of Biosystem Shahrekord University Shahrekord Iran

**Keywords:** ferrioxamine B, molecular dynamics simulation, plasma‐activated water, *Salmonella*

## Abstract

*Salmonella* remains one of the most prevalent foodborne pathogens worldwide, significantly contributing to gastrointestinal illnesses. Therefore, it is crucial to employ methods that can effectively reduce or eliminate the presence of this harmful pathogen in agricultural products. The current study employed a continuous production system of plasma‐activated water (PAW) to control and eliminate *Salmonella* in almonds. The study examined the impact of key operational parameters, including water flow rate, gas mixture ratio (air:argon), and the duration of applying PAW. Results demonstrated that plasma influenced water flow rate, had the greatest effect in inactivating *salmonella*, followed by the combined gas ratio and the PAW application time, respectively. PAW can affect *Salmonella* viability through damage to DNA, membrane lipids, and protein structure destruction. Among all the mechanisms of PAW effect on *Salmonella*, the impact of free radicals on ferrioxamine B was investigated as a hypothesis for the effect on *Salmonella* viability by MD simulation. By utilizing the Molecular Dynamics simulation method, the effect of three free radicals, namely nitrate (NO_3_), hydrogen peroxide (H_2_O_2_), and oxygen (O_2_), on the ferrioxamine B receptor of *Salmonella* was investigated. The results indicated that H_2_O_2_ had a more significant modulatory effect of the receptor, as evidenced by the changes in the radius of gyration and RMSF of ferrioxamine B. Additionally, H_2_O_2_ demonstrated more extensive effects on the average intermolecular hydrogen bonds than the other two free radicals. The computational modeling suggests that ROS in PAW may compromise bacterial viability by structural destabilization of the ferrioxamine B receptor, thereby supporting a plausible molecular mechanism for the observed antimicrobial efficacy leading to its destruction.

## Graphical abstract

Plasma‐activated water (PAW) was generated using a cold plasma jet system to eliminate *Salmonella* from almonds. Response surface methodology (RSM) optimized three operational parameters: argon‐to‐air ratio, water flow rate (*Q*), and PAW application time (*T*). Under optimal conditions (argon 100%, *Q* = 0.5 mL/min, *T* = 1.16 min), a maximum 3.25 log reduction of *Salmonella* was achieved. Concurrently, molecular dynamics (MD) simulations revealed that reactive oxygen/nitrogen species (ROS/RNS), particularly H_2_O_2_, destabilize the ferrioxamine B receptor of *Salmonella*, providing a molecular mechanism for the observed antimicrobial efficacy.

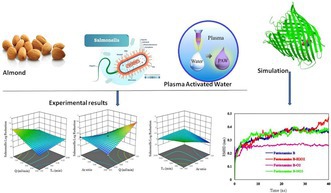

## 1 Introduction

*Salmonella* is a common pathogen, responsible for 80 million cases of gastrointestinal illness worldwide each year (Majowicz et al. 2010). *Salmonella* infections are typically contracted through contaminated food, particularly vegetables, green leafy produce, fruits, and nuts (Bennett et al. 2015; De Oliveira Elias et al. 2018; Reddy et al. 2016).

Researchers have been exploring new technologies to control microbial pathogens in agricultural products. One promising technology is non‐thermal plasma, although there are limitations to using it for treating the surface of products (Guo et al. 2021). As a result, some researchers have turned to plasma‐activated water (PAW), which exhibits high efficacy in controlling microbial contamination (Aarabi et al. 2025, 2024; Han et al. 2022). Research has established that PAW primarily contains reactive oxygen species (ROS) and reactive nitrogen species (RNS), encompassing hydroxyl radicals (•OH), hydrogen peroxide (H_2_O_2_), singlet oxygen (^1^O_2_), superoxide anions (O_2_•^−^), ozone (O_3_), nitrate (NO_3_^−^), nitrite (NO_2_^−^), peroxynitrite (ONOO^−^), nitric oxide radicals (•NO), and ammonia (NH_3_) (Aarabi et al. 2025, 2024; Guo et al. 2021; Samukawa et al. 2012).

Iron is a crucial micronutrient for bacterial growth. Bacteria have developed various methods to absorb iron, the most common being siderophore‐mediated iron absorption (Llamas et al. 2006; Saldaña‐Ahuactzi and Knodler 2022). Siderophores are produced by microorganisms when iron is scarce, and they work alongside receptor molecules to transfer iron from the environment to bacteria (Kraemer 2004). Additionally, siderophores are among the compounds that protect the plant immune system against a wide range of plant pathogens (Karplus and McCammon 2002). Therefore, disrupting iron acquisition via siderophore receptors, such as ferrioxamine B in *Salmonella*, represents a potential antimicrobial strategy. Molecular dynamics (MD) simulation is a powerful tool to investigate such interactions at the atomic level, providing insights into biomolecular stability and conformational changes (Karplus and McCammon 2002). These computational methods enable quantitative assessment of conformational flexibility across distinct domains of biomolecules through time‐resolved atomic trajectory analysis (Li et al. 2014).

This study had two primary objectives: (1) to experimentally determine the optimal parameters (water flow rate, gas ratio, application time) for PAW to reduce *Salmonella* on almonds using RSM, and (2) to computationally investigate, via MD simulations, the molecular‐level interaction of key PAW‐derived radicals (H_2_O_2_, O_2_, NO_3_^−^) with the ferrioxamine B siderophore receptor in *Salmonella*. We further aimed to investigate whether a meaningful connection exists between these experimental and computational findings. Studies have shown that the main active ingredients of PAW are ROS and RNS. The main components of ROS include hydroxyl radicals, hydrogen peroxide, singlet oxygen, superoxide anions, and ozone, while RNS mainly include nitrate, nitrite, peroxynitrite, nitric oxide radical, and ammonia. Among them, the long‐lived active species are hydrogen peroxide, nitrate, nitrite, and oxygen radical. Therefore, considering the longer lifetime of these radicals in PAW and the laboratory limitations for measuring their concentrations, these radicals were chosen (Guo et al. 2021). A key strength of this work is the integration of a benchtop statistically‐optimized engineering process (RSM) with high‐resolution atomistic computational modeling (MD) to propose a mechanism—a powerful and non‐trivial approach.

## 2 Materials and Methods

### 2.1 Plasma‐Activated Water Production System

The plasma properties are affected by various factors, including the plasma production system's geometry, the electrodes' placement and shape, the applied frequency and voltage, the electric current, and the gas properties (Esmaeili et al. 2023). The system consisted of an AC power supply, tungsten electrodes, a 3‐mm gap distance, a ceramic dielectric, variable voltage up to 25 kV, variable frequency from 10 to 35 kHz, and a power output of 500 W. Plasma generation utilizes various gases, including argon (Ar), nitrogen (N_2_), and oxygen (O_2_), and their combinations. Air, readily available and inexpensive, contains nitrogen and oxygen, making it an effective source for plasma production.

**FIGURE 1:**
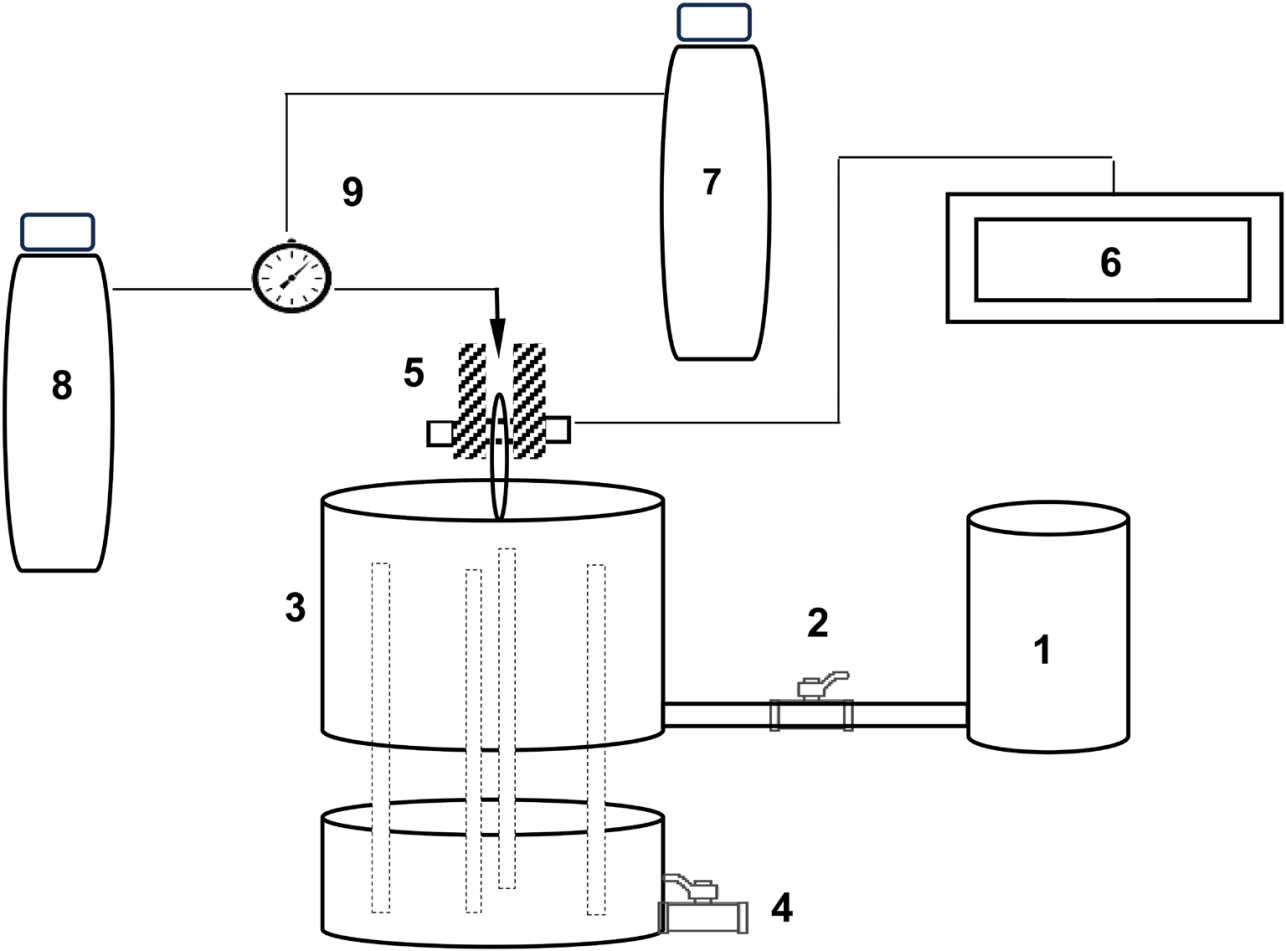
Schematic of the plasma‐activated water (PAW) generation system: (1) water reservoir inlet, (2) precision flow control valve, (3) cylindrical treatment chamber, (4) outlet conduit for plasma‐treated effluent, (5) integrated dielectric barrier discharge (DBD) plasma reactor with spray nozzle assembly, (6) high‐voltage plasma power supply, (7) argon (Ar) gas source, (8) compressed air supply, and (9) gas mixing ratio controller.

In this system, the incoming water is directed into the treatment chamber via a one‐way valve, where it undergoes plasma discharge exposure. The inlet and outlet flow rates were precisely controlled and adjusted to achieve stable flow conditions. This optimization ensured optimal exposure time for effective plasma‐based water treatment while maintaining uninterrupted flow continuity. The hydrodynamic parameters of the system were systematically tuned until desired performance was attained (refer to Figure 1 for system details).

The generation of PAW was achieved utilizing an atmospheric‐pressure dielectric barrier discharge (DBD) cold plasma jet system. The designed system allowed for a continuous water flow into the treatment container, where it was exposed to the plasma before continuous discharge through the outlet port. The flow was controlled using a one‐way valve to ensure stable flow and uninterrupted plasma treatment. Various gas mixtures (argon and air) and water flow rates were used to produce PAW for *Salmonella* treatment. The effectiveness of PAW on *Salmonella* was studied with varying application times of 1, 10, and 20 min.

### 2.2 Preparation of Samples, Culture, and Inoculation of Bacteria

The California almond specialist breed guidelines were followed for the cultivation, inoculation, and reporting process (Almond Board of California 2014). Almond kernels (400 g, Maami variety, Chaharmahal and Bakhtiari provinces, Iran) were exposed to UV irradiation (254 nm) to ensure microbial safety. A 25‐mL bacterial suspension was required to inoculate the kernels. The bacterial strain was cultured on tryptic soy agar plates and incubated at 35°C for 24 ± 2 h. The cells were transferred to Tryptic Soy Broth (10 mL) and incubated at 35°C for 24 ± 2 h.

A loopful of the broth culture was then transferred to Tryptic Soy Broth (10 mL) and incubated at 35°C for 18 ± 2 h. The bacteria were then cultivated (1 mL per plate) on 5 large tryptic soy agar plates (150 × 15 mm) and grown for 24 ± 2 h at 35°C. 0.1% peptone (6 mL) was added to each plate, and the sterile spreader was used to loosen the bacterial surface. The cells were collected using a sterile pipette in a sterile chamber. The inoculum was prepared by mixing 25 mL of the broth culture and stirring thoroughly it for 1 min with a magnetic stirrer. The inoculum was then added to the almonds, which were uniformly distributed on 4 layers of filter paper and allowed to dry for 24 h at 24°C ± 2°C.

The inoculated almonds were collected in sterile polyethylene storage containers and stored at a temperature of 4°C ± 1°C. After 24 h, the bacterial colonies were counted in the samples. According to the experimental plan, the almonds were subjected to PAW to evaluate the reduction of *Salmonella* bacteria.

### 2.3 Recovery and Counting of Inoculated Microorganisms

After applying PAW, almond samples were combined with Tryptic Soy Broth at a ratio of 1:2 (25 g of almonds to 50 mL of TSB) at room temperature. The mixture was then blended in a bag with a paddle mixer for 2 min and allowed to stand for 3–5 min. Serial dilutions were prepared in Butterfield's phosphate buffer, and 0.1 mL of each diluted solution was cultured on tryptic soy agar plates. Following 48‐h incubation at 35°C, bacterial enumeration was performed via colony counting to quantify viable microbial populations.

### 2.4 The Method of Analysis, Modeling, and Optimization of the Experiment

The most effective conditions for eradicating *Salmonella* from the samples were identified by using the response surface methodology (RSM). The response surface method encompasses a series of mathematical and statistical techniques employed to model and optimize processes influenced by numerous variables (Hosseinzadeh Samani et al. 2020). The systematic procedure for establishing response‐variable relationships through this methodology involves the following key phases:

1. Identifying the independent variables, their operational levels, and selecting an appropriate test plan.

2. Conducting prediction and accuracy investigations.

3. Generating response surface and contour plots from the derived model equation to visualize variable interactions and linear relationships.

The optimal solution was derived through analytical resolution of the system equation (Hosseinzadeh Samani et al. 2020):

(1) $$Y_{i}=\beta_{0}+\sum {Β}_{i}\;X_{i}+\sum \beta_{\mathrm{ii}}\;{X_{i}}^{2}+\sum \beta_{\mathrm{ii}}\;X_{i}\;X_{\mathrm{ij}}+\varepsilon$$

where *β*_*0*_, *β*_*i*_, and *β*_*ii*_ are constant coefficients, *X*_*i*_ and *X*_*ij*_ are independent variables in the process, and *ε* is a random error. The operational ranges for independent variables were established based on preliminary studies and process constraints, as detailed in Table 1 (Hosseinzadeh Samani et al. 2020).

**TABLE 1:** The selected levels of independent variables in the Response Surface Methodology (RSM) design for *Salmonella* bacteria.

Independent variable	Range of level		
	−1	0	1
Water flow rate (mL/min)	0.5	1	1.5
Ar ratio (air + argon)	0	0.5	1
PAW application time (min)	1	10	20

### 2.5 Molecular Dynamics Simulation and In Silico Phase Study

To study how PAW affects *Salmonella* bacteria, we conducted in silico molecular dynamics simulations to observe the changes in the ferrioxamine B receptor.

#### 2.5.1 System Setup

The simulation system was constructed using Packmol by placing ferrioxamine B and the selected radicals at specific concentrations in a water box. Three radicals (H_2_O_2_, O_2_, and NO_3_^−^) were examined, and each system was saved in PDB format. The output data from GROMACS were analyzed using Microsoft Excel; results were visualized as two‐dimensional plots and summarized in tables as mean ± SD. Molecular structures of NO_3_^−^, H_2_O_2_, and O_2_ were obtained from PubChem (CIDs 943, 784, and 977, respectively). As their three‐dimensional coordinates were not available in the database, they were modeled using Chem3D 18.0. The ferrioxamine B [M + Fe‐2H] structure was also obtained from PubChem (CID: 64173771).

#### 2.5.2 Simulation Parameters

Each system was solvated in a box with TIP4P water molecules. Energy minimization was performed using the steepest descent and conjugate gradient algorithms. Following minimization, position‐restrained NVT equilibration was conducted at 100°K for 500 ps, followed by NPT equilibration at 100°K and 1 bar for 500 ps. Berendsen dynamics were used for temperature control during equilibration. The temperature was then gradually increased from 0°K to 300°K, with velocities reassigned at each step according to the Maxwell–Boltzmann distribution, and equilibrated for an additional 300 ps. Production MD simulations were performed for 40 ns at 300°K with a 2‐fs time step. The Nose‐Hoover thermostat and isotropic Monte Carlo barostat maintained the temperature at 300°K and pressure at 1 atm. Lennard–Jones potential and Particle Mesh Ewald (PME) methods were used for van der Waals and electrostatic interactions, respectively.

#### 2.5.3 Trajectory Analysis

Energy minimization of the compounds was performed using UCSF Chimera 1.13.1, and the resulting files were saved in PDB format. MD simulations were conducted using GROMACS 5.1.4. Topology files for the radicals and ferrioxamine B were generated using the ACPYPE tool, as these structures lacked recognizable protein/nucleic acid topologies in GROMACS. The AMBER force field was applied for all simulations. Radical‐receptor interactions were examined by docking (AutoDock 4.2) using radical structures generated with ArgusLab and the ferrioxamine B receptor structure obtained from RCSB PDB. Molecular interactions were visualized using PyMOL.

## 3 Results and Discussion

### 3.1 The Effect of Plasma‐Activated Water on *Salmonella* Bacteria

The experimental design employed a Box–Behnken response surface methodology (RSM) to systematically evaluate three critical independent variables: air‐to‐gas ratio (air + argon), plasma water flow rate, and PAW application time. A stepwise analysis of variance was conducted, and the results are presented in Table 2. The variance analysis reveals the main and reciprocal impacts of activated water application time and flow rate of water on the reduction of *Salmonella* in almonds under the influence of plasma and air‐to‐gas ratio (air + argon).

**TABLE 2:** Statistical analysis results of quadratic response surface model for logarithmic reduction of *Salmonella* bacteria.

Source	Sum of squares	df	Mean square	*F*‐value	*p*
Model	3.49	8	0.4365	395.81	< 0.0001
A‐Q	0.8321	1	0.8321	754.43	< 0.0001
B‐Ar	0.7260	1	0.7260	658.29	< 0.0001
C‐Tw	0.0703	1	0.0703	63.75	0.0002
AB	1.16	1	1.16	1047.82	< 0.0001
AC	0.3660	1	0.3660	331.88	< 0.0001
BC	0.3249	1	0.3249	294.59	< 0.0001
*A* ^2^	0.0101	1	0.0101	9.15	0.0233
*B* ^2^	0.0061	1	0.0061	5.49	0.0576
Residual	0.0066	6	0.0011		
Lack of fit	0.0060	4	0.0015	5.01	0.1731
Pure error	0.0006	2	0.0003		
Cor total	3.50	14			

According to Table 2, all factors except the quadratic term of application time (C^2^) significantly affected the reduction of *Salmonella*. The model has a coefficient of determination of 0.998 (*R* ^2^) and a standard error (Std. Dev.) of 0.03, indicating that it is highly accurate.

The experimental data yielded the following coded Equation (2) to quantify the effect of the independent variables on the reduction of *Salmonella* bacteria.

(2) $$\begin{array}{c} \text{Salmonella}=-2.68+0.3225A-0.3012B-0.0937C-0.5375\mathrm{AB}\\ \;-0.3025\mathrm{AC}+0.2850\mathrm{BC}+0.0521\;A^{2}-0.0404\;B^{2} \end{array}$$

Based on the coefficient values in Equation (2), it can be concluded that the water flow rate influenced by plasma (A) is the most effective variable in reducing *Salmonella*. The combined gas ratio (B) and activated water application time (C) follow. Negative coefficients indicate an inverse relationship between the independent variable and the rate of *Salmonella* reduction. This means that as the factor increases, the rate of reduction decreases and vice versa.

**FIGURE 2:**
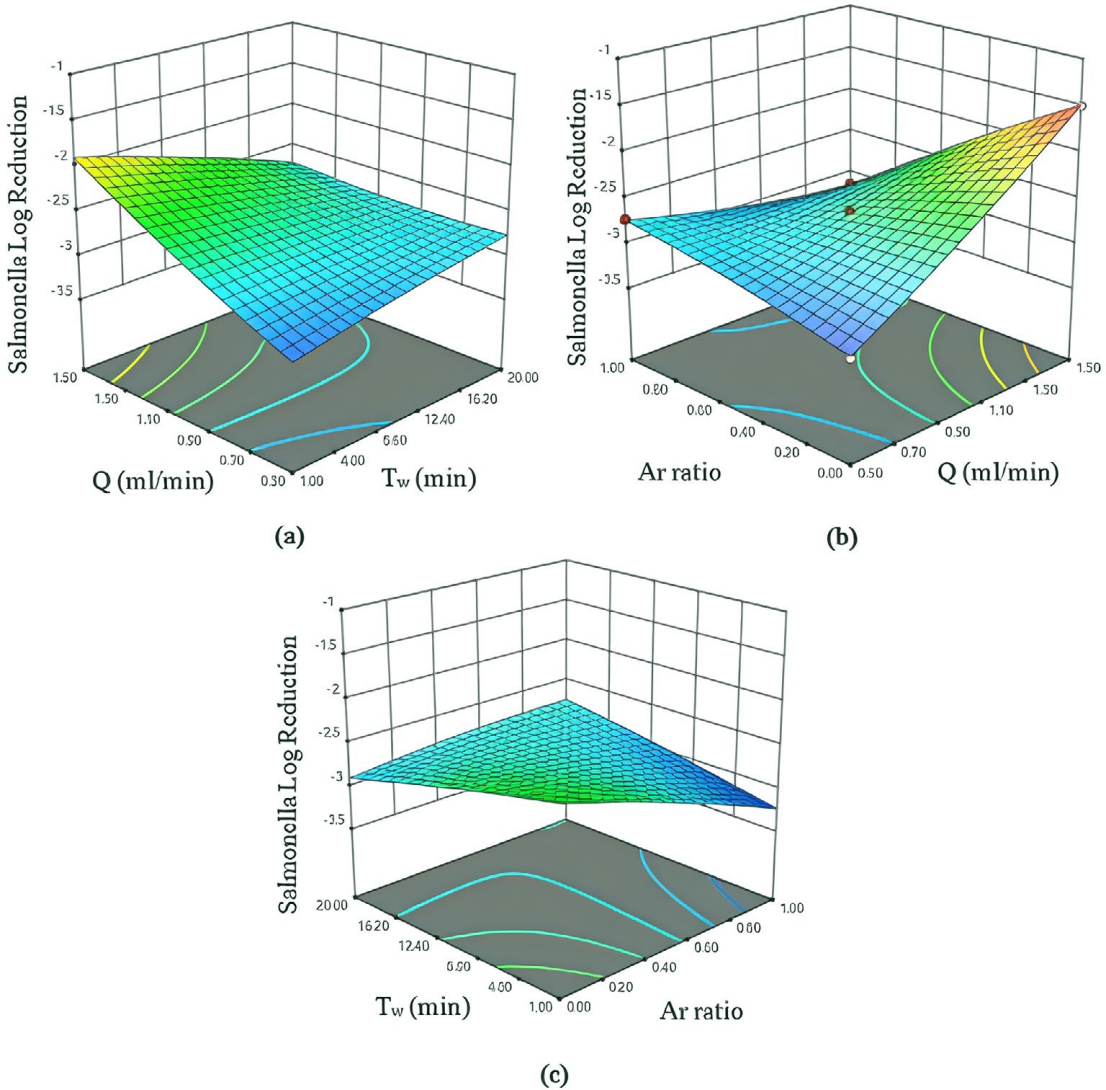
The logarithmic reduction of *Salmonella* was shown as a function of: (a) the ratio of air and argon composition and the flow rate of plasma‐activated water (*Q*); (b) the flow rate of plasma‐activated water (*Q*) and the application time of activated water (*T*_*W*_); and (c) the ratio of the combination of argon and air and the application time of activated water (*T*_*W*_).

In Figure 2a, the combination of air with argon and PAW flow was tested to determine their effect on reducing *Salmonella*. The results showed that reducing the flow of water under plasma treatment conditions from 1.5 to 1 mL/min increased the reduction of *Salmonella* from 1.5 to 2.5 log units. Additionally, decreasing the water flow rate from 1 to 0.5 mL/min increased the reduction of *Salmonella* from 2.5 log units to about 3.5.

Figure 2b examined the impact of PAW flow rate and PAW application time in reducing *Salmonella* bacteria. The findings revealed that the water flow under plasma treatment conditions had a greater effect in reducing *Salmonella* than when activated water was applied. PAW application time was found to be a significant factor in reducing *Salmonella*. By increasing the time of application from 1 to 10 min, the amount of *Salmonella* reduction increased from 3 log units to 3.5 log units. However, increasing the PAW application time from 10 to 20 min showed a constant trend.

An increase in the flow rate reduced the exposure time of water to plasma, while a decrease in the flow rate increased the plasma‐water contact time. Prolonged plasma treatment of water generates free radicals, positive/negative ions, and reactive species (ROS/RNS) that diffuse into the aqueous phase. As the concentration of these highly reactive species rises, water's ability to act as a reducing and oxidizing agent is enhanced, and the acidity of the environment increases due to a decrease in pH. Treatment of *Salmonella*‐contaminated almonds with PAW resulted in significant bacterial reduction.

Prolonged exposure of samples to activated water increases the likelihood of reactions and damage caused by ions and free radicals, leading to enhanced control of *Salmonella* bacteria. Figure 2c illustrates how varying the proportion of air relative to the total gas mixture (air plus argon), along with the duration of activated water exposure, influences the decline in *Salmonella* populations. As the proportion of argon in the gas mixture increased from 0% to 50%, *Salmonella* reduction increased by 0.25 log units. Furthermore, increasing the argon proportion from 50% to 100% further reduced *Salmonella* by 0.34 log units.

The use of various gases to generate plasma results in the formation of highly reactive nitrogen and oxygen species (RNS and ROS), which can either interact with the water surface or diffuse into it, initiating a series of complex chemical reactions. The generation of free radicals and highly reactive chemical species plays a key role in the antimicrobial action of plasma against *Salmonella* bacteria.

When applying PAW to samples, as the levels of free radicals and reactive species rise, more damage occurs to the bacteria, viruses, and toxins present in the samples. The type and concentration of free radicals each gas produces affect their impact. Water activated by air plasma contains higher levels of singlet oxygen radicals, nitric oxide, nitrate, and nitrite compared to that treated with argon plasma (Takamatsu et al. 2012, 2015). Conversely, water activated with argon plasma has more free radicals of positive single hydrogen, hydroxyl, and hydrogen peroxide than water activated with air plasma (Takamatsu et al. 2012, 2015). Introducing an optimal level of reactive nitrogen and oxygen species (RNS and ROS) into PAW disrupts the structure of *Salmonella* and thus inactivates the bacteria in the samples.

### 3.2 Optimization

**FIGURE 3:**
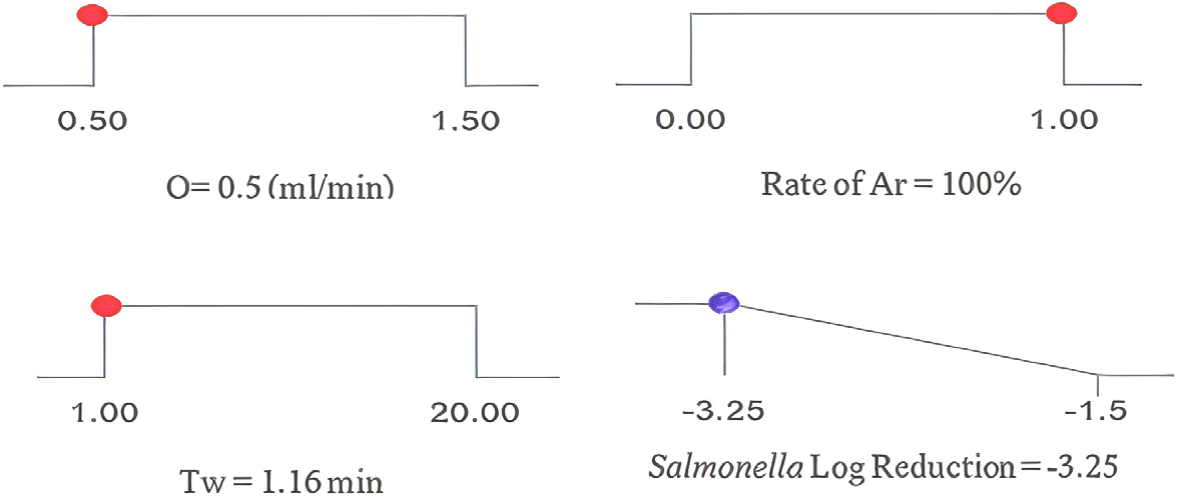
Process optimization based on response surface model for plasma‐activated water system.

Figure 3 displays the optimization results of the response surface method, showing the maximum reduction of *Salmonella* bacteria. The system setup and operational parameters for generating and utilizing PAW were fine‐tuned to reach optimal performance levels. These values include a water flow rate of 0.5 mL/min, 100% argon gas (0% air), and a PAW application time of 1.16 min. A 3.25 log unit reduction in *Salmonella* was achieved with these values. To verify the identified optimal conditions, the reduction rate was evaluated in a controlled laboratory setting under consistent parameters. The resulting reduction rate was 3.2 log units, indicating a minimal gap between the predicted and actual optimum results, highlighting the accuracy of the optimization method.

### 3.3 In Silico Molecular Dynamics Simulation

An experiment was conducted to study the impact of PAW on the structure of *Salmonella* under optimal conditions. To understand the molecular mechanism, MD simulations were conducted focusing on key radicals identified from the PAW chemistry. The PAW production system (as shown in Figure 2) was used with 100% argon gas and a water flow rate of 0.5 mL/min. The concentration of three target free radicals was measured by titration: approximately 4 ppm for H_2_O_2_, 20 ppm for O_2_, and 5 ppm for NO_3_.

In the simulation box, 10,000 water molecules were used, and according to the simulation conditions, the molar concentration of NO_3_ was 6.44 × 10^−7^ M, H_2_O_2_ was 1.47 × 10^−6^ M, and the molar concentration of O_2_ was 6.56 × 10^−6^ M. These free radicals were then placed in a box of water with *Salmonella*, and a computational analysis of PAW containing nitrate radicals (NO_3_), hydrogen peroxide (H_2_O_2_), and oxygen (O_2_) was performed for 40 ns in the presence of ferrioxamine B receptor.

**FIGURE 4:**
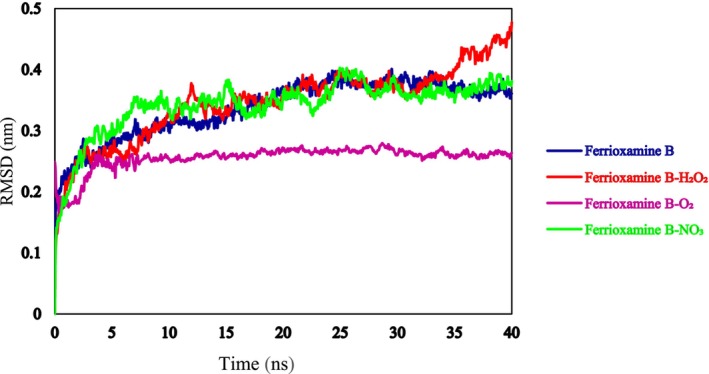
Changes (RMSD) between *Salmonella* particles during simulation of molecular dynamics in the presence of plasma‐activated water.

Figure 4 displays the changes (Root Mean Square Deviation) of RMSD between the alpha carbon (interparticle interactions) in the ferrioxamine B receptor during 40 ns of molecular dynamics simulation in water. The RMSD value reflects alterations in the protein's tertiary structure during the simulation and may also provide insights into its charge distribution (Zhang and Lazim 2017). An increase in RMSD values suggests enhanced structural instability of the compound when exposed to the specified factors (Bhat et al. 2020; Doss et al. 2012). As shown in Figure 4, O_2_ induced the lowest RMSD, followed by NO_3_^−^, while H_2_O_2_ caused the greatest conformational change and structural destabilization of the ferrioxamine B receptor. According to Table 3, the average RMSD values for the three radicals O_2_, NO_3_, and H_2_O_2_ are respectively 0.255 ± 0.020, 0.341 ± 0.036, and 0.345 ± 0.061. H_2_O_2_ had the greatest effect, and O_2_ had the smallest effect on RMSD.

**TABLE 3:** Mean and standard deviation of RMSD, RMSF, and Rg (after 40 ns).

System	RMSD (nm)	RMSF (nm)	Rg (nm)
NO_3_^−^	0.341 ± 0.046	0.081 ± 0.049	2.606 ± 0.013
H_2_O_2_	0.345 ± 0.061	0.141 ± 0.115	2.613 ± 0.019
O_2_	0.255 ± 0.020	0.120 ± 0.097	2.55 ± 0.006

**FIGURE 5:**
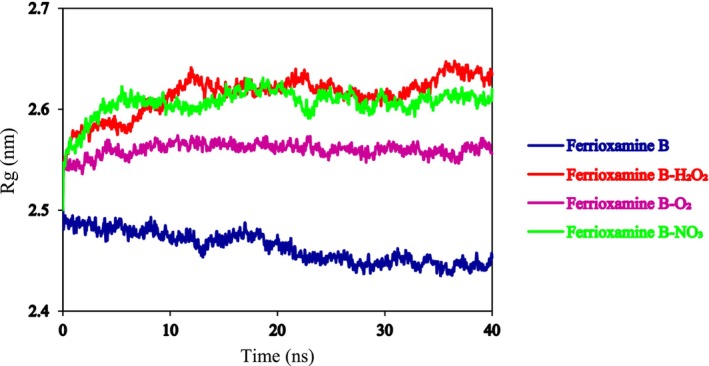
Radius of rotation (Rg) of ferrioxamine B during simulation of molecular dynamics in the presence of plasma‐activated water.

The diagram in Figure 5 displays the alterations in ferrioxamine B's radius of gyration (Rg) during simulation of molecular dynamics in the presence of PAW. The Rg is a parameter commonly used to assess the degree of compactness in proteins and molecular compounds. A structurally stable compound is capable of preserving its Rg, even under conditions that induce unfolding. During the simulation process, the Rg value may increase (McGibbon et al. 2015). A smaller radius of gyration indicates a more compact molecular structure, which is generally associated with greater thermal stability (Bhat et al. 2020). As shown in Table 3, shifts in ferrioxamine B's Rg in the presence of H_2_O_2_ are 2.631 ± 0.019, which has a more significant effect on the receptor's structure compared to the other two free radicals. The values for O_2_ and NO_3_ were 2.55 ± 0.006 and 2.606 ± 0.013, respectively, indicating that O_2_ had the least impact on the receptor.

**FIGURE 6:**
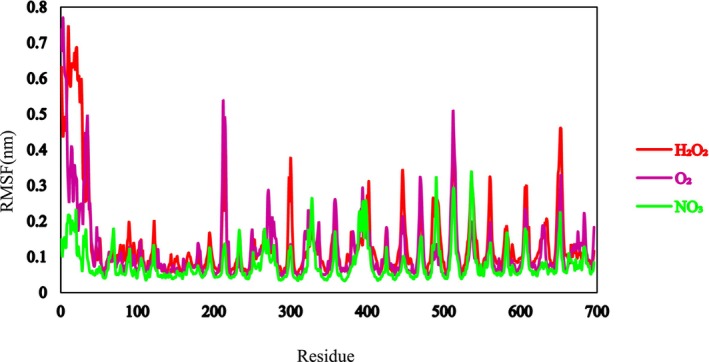
Changes related to the RMSF of the amino acid units of the ferrioxamine B receptor in 40 ns of simulation in the presence of plasma‐activated water.

Figure 6 shows the changes in Root Mean Square Fluctuation (RMSF) of amino acid residues in the ferrioxamine B receptor during a 40‐ns simulation in PAW. The RMSF analysis quantifies how flexible the polypeptide backbone is by analyzing shifts in alpha carbon positions at each residue over time. It evaluates the flexibility of individual residues or atoms relative to their initial positions at the beginning of the simulation. Loops typically exhibit the highest RMSF values and are therefore useful for studying the impact of mutations or other perturbations on protein flexibility. Protein unfolding typically leads to an increase in both residue entropy and RMSF, whereas a reduction in RMSF reflects enhanced structural stability. As shown in Figure 6 and Table 3, the average RMSF value of ferrioxamine B in the presence of H_2_O_2_ was 0.141 ± 0.115, indicating greater conformational changes compared to the other radicals. The values for O_2_ and NO_3_ were 0.120 ± 0.097 and 0.081 ± 0.049, respectively, demonstrating that NO_3_ had the lowest impact on the receptor.

**FIGURE 7:**
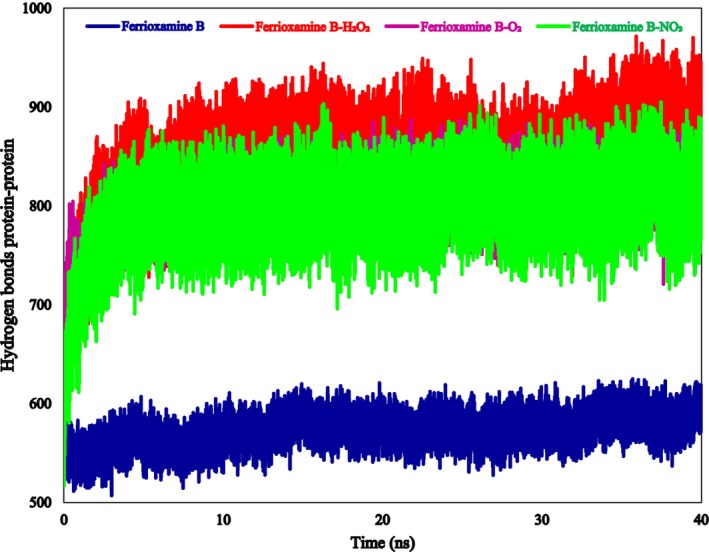
Intermolecular hydrogen bond interactions in ferrioxamine B at 40 ns.

**FIGURE 8:**
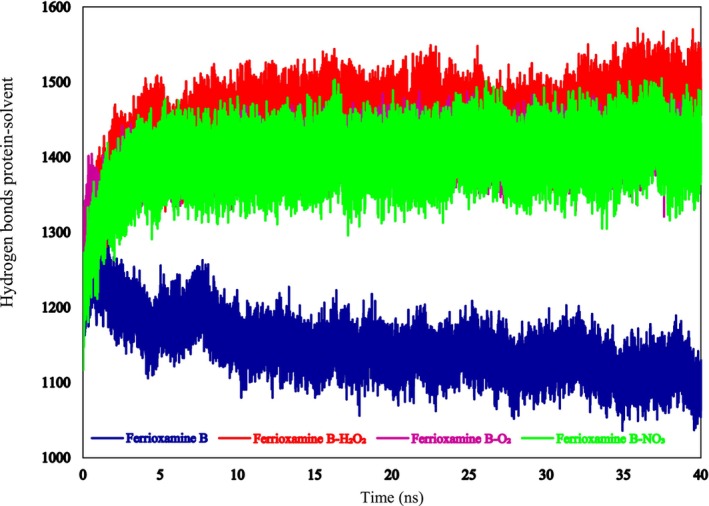
Hydrogen bond interactions between ferrioxamine B and solvent in 40 ns.

The study of intermolecular hydrogen bonds in ferrioxamine B revealed that H_2_O_2_ had a greater impact compared to NO_3_ and O_2_, as demonstrated in Figure 7 and Table 3. H_2_O_2_ increased the number of hydrogen bonds in ferrioxamine B, resulting in alterations to the receptor's structure. H_2_O_2_ forms more hydrogen bonds with PAW and ferrioxamine B, which can cause further changes in the solubility of the compound and thereby create more unstable structures. Figure 8 shows that H_2_O_2_ has a greater action in hydrogen bonding compared to NO_3_ and O_2_ when interacting with the solvent.

Based on the data presented in Table 4, the interactions of O_2_ and NO_3_ were nearly identical. Additionally, H_2_O_2_ showed the strongest hydrogen bonding in this scenario. Furthermore, the software has estimated the total binding free energy (∆G bind, kcal/mol) as follows:

$$\text{∆G bind}=G\;\text{complex}-\left(\text{Gferrioxamin}\;B+G\;\text{radical}\right)$$

**TABLE 4:** Average number of hydrogen bonds during 40 ns.

System	Number of hydrogen bonds	
	Ferrioxamin B–free radical	Ferrioxamin B–ferrioxamine B
O_2_	1405.42 ± 26.56	607.309 ± 10.92
H_2_O_2_	1446.908 ± 44.29	1446.908 ± 44.29
NO_3_	1401.117 ± 3 9.33	602.63 ± 11.49

According to Table 5, O_2_ showed the most negative binding energy (−8.23 ± 0.74 kcal/mol), indicating the strongest interaction with ferrioxamine B. H_2_O_2_ and NO_3_^−^ exhibited higher binding energies (−6.33 ± 0.74 kcal/mol), suggesting weaker binding. The stronger binding of O_2_ to ferrioxamine B may potentially influence *Salmonella* structure and function.

**TABLE 5:** Total binding free energy between ferrioxamine B and free radicals.

System	Kcal/mol (∆*G* bind)
O_2_	−8.23 ± 0.74
H_2_O_2_	−6.33 ± 0.74
NO_3_	−6.33 ± 0.74

Table 6 presents the average values of kinetic energy, potential energy, total energy, density, temperature, and enthalpy of the ferrioxamine B system during 40 ns of simulation in PAW containing the respective radicals. Table 6 illustrates that the presence of all three reactive species resulted in a notable rise in the system's potential energy when interacting with ferrioxamine B, suggesting a structural or energetic impact on the protein. The simulation of ferrioxamine B in the presence of NO_3_^−^ shows a lower temperature, as seen in Table 6, suggesting that this active species had a lesser impact on the receptor than the other two species.

**TABLE 6:** Values of total, potential, kinetic, and enthalpy energies, along with temperature and density, throughout the simulation period.

Energy	O_2_	H_2_O_2_	NO_3_
Potential energy (KJ/mol)	−9582037.06 ± 269594.28	−6836142.21 ± 281267.61	−8044731.73 ± 335438.88
Kinetic energy (KJ/mol)	1731463.73 ± 4384.97	122425.08 ± 305923.89	1441388.79 ± 365173.34
Total energy (KJ/mol)	7850381.87 ± 268311.13 —	−5611888.13 ± 586305.01	−6603342.94 ± 699562.10
Temperature (°K)	300.17 ± 0.76	300.06 ± 74.98	300.75 ± 76.19
Density (kg/m^3^)	982.82 ± 7.84	945.75 ± 2.52	943.84 ± 2.71
Enthalpy (KJ/mol)	−7811239.41 ± 5830.07	−5611576.90 ± 586304.64	−6602976.55 ± 699561.63

**FIGURE 9:**
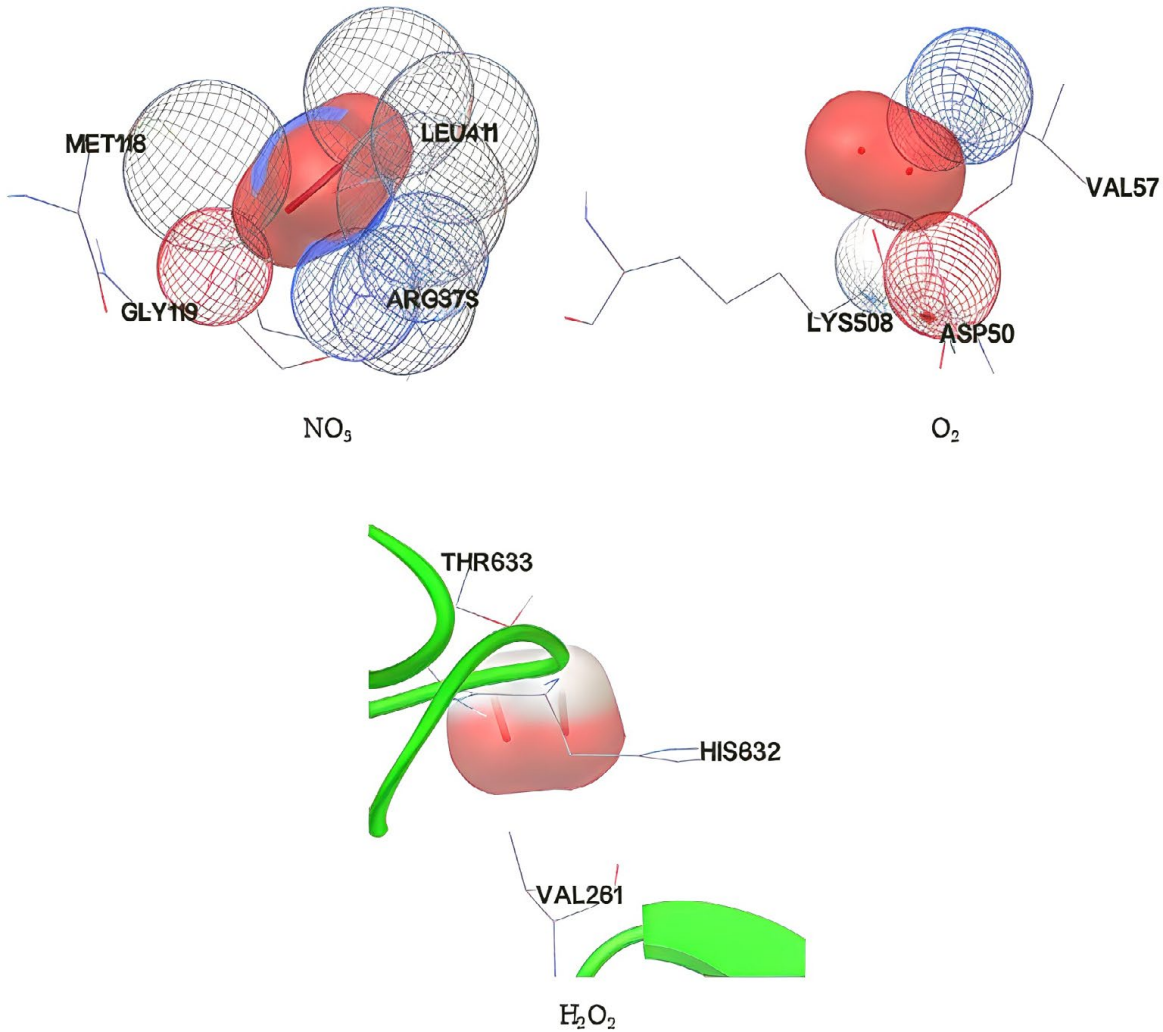
The mode of interaction and the atoms involved in free radicals (O_2_, NO_3_, and H_2_O_2_) with the ferrioxamine B in the shortest distance during the simulation.

In order to find out how the free radical binds to the compound of the ferrioxamine B receptor, the three‐dimensional structure of the complex was determined at the minimum intermolecular distance. The spatial arrangement and binding mode are illustrated in Figure 9. As shown in this figure, the O_2_ radical binds to amino acids valine 57 (Val 57), lysine 508 (Lys 508), and aspartic acid 50 (Asp 50) of the ferrioxamine B receptor.

Additionally, the simulation results indicate that the NO_3_ interacts with methionine 118 (Met118), glycine 119 (Gly119), leucine 411 (Leu411), and arginine 373 (Arg373).

Similarly, the simulation results demonstrate that H_2_O_2_ interacts with valine 261 (Val261), histidine 632 (His632), and threonine 633 (Thr633) within the ferrioxamine B receptor.

Understanding the interaction of these reactive species with receptor structures and their impact on specific amino acids reveals how PAW compounds influence the structural integrity of the ferrioxamine B receptor, ultimately contributing to the inhibition of *Salmonella*.

It is important to note that these images, generated using Autodock 4.2, illustrate the interaction between reactive species and the ferrioxamine B receptor, representing the most favorable binding conformations observed.

## 4 Conclusion

This study investigated the use of PAW to control and eliminate *Salmonella* in almonds. Reducing the water flow rate during plasma treatment significantly enhanced bacterial reduction. Subsequently, the air:argon gas ratio (air + argon) and PAW exposure time were examined. The study reported a decrease of 1.5 to 3.5 log units in *Salmonella* in almonds under different treatments using PAW. The optimal conditions for reducing *Salmonella*were a water flow rate of 0.5 mL/min, 100% argon (0% air), and a PAW application time of 1.16 min. PAW can affect *Salmonella* viability through multiple mechanisms, including damage to DNA, membrane lipids, and protein structure. Among all the mechanisms of PAW effect on *Salmonella*, the effect of free radicals on the ferrioxamine B was investigated as a hypothesis for the effect on *Salmonella* viability using MD simulation. The MD simulation study also found that the O_2_ free radical reduced the RMSD compared to NO_3_ and H_2_O_2_. This indicates O_2_ that inhibit the activity of the membrane protein receptor of the ferrioxamine B less compared to the two other, while NO_3_ has the highest effectiveness. The Rgof ferrioxamine B for H_2_O_2_ and O_2_ was almost the same but lowest for NO_3_. Thus, the receptor is likely to exhibit greater thermal stability when exposed to two H_2_O_2_ radicals and O_2_. In summary, the findings indicate that free radicals present in PAW have the potential to alter the structural integrity of the ferrioxamine B receptor membrane. This study provides a molecular rationale for the observed efficacy of PAW, moving beyond empirical observations to a testable hypothesis about its mode of action.

## Author Contributions

**Bahram Hosseinzadeh Samani:** writing – review and editing, validation, methodology, project administration, conceptualization, supervision. **Sajad Rostami:** writing – review and editing, validation, methodology, project administration, conceptualization, funding acquisition, data curation, supervision. **Mohammad Javad Aarabi:** writing – original draft, investigation, software, visualization, data curation, resources, formal analysis.

## Funding

This research was funded by the Research Council of Shahrekord University, Grant no. 97GRN31M1007.

## Conflicts of Interest

The authors declare no conflicts of interest.

## Data Availability

The data that support the findings of this study are available from the corresponding author upon reasonable request.
